# Research progress on NUSAP1 and its role in digestive system neoplasms

**DOI:** 10.3389/fonc.2025.1582361

**Published:** 2025-06-04

**Authors:** Shangui Fu, Yuting Zhuo, Tao Yu

**Affiliations:** ^1^ Department of General Surgery, Jiujiang University Affiliated Hospital, Jiujiang, Jiangxi, China; ^2^ The Second School of Clinical Medicine, Jiangxi Medical College, Nanchang University, Nanchang, Jiangxi, China

**Keywords:** NUSAP1, digestive system neoplasms, signaling pathway, cell cycle, microtubule

## Abstract

Nucleolar spindle-associated protein 1 (NUSAP1) is a microtubule-binding protein critical in the mitotic cell cycle. Its primary functions encompass maintaining microtubule stability, facilitating spindle assembly, regulating chromosome alignment, and modulating multiple signaling pathways. The incidence and mortality rates of digestive system neoplasms are among the highest of all malignant tumors. Therefore, identifying effective biological targets for targeted cancer control and treatment strategies is critical. Recent studies have demonstrated that NUSAP1 is highly expressed in various malignant tumors of the digestive system and plays a pivotal role in the initiation, progression, treatment, and prognosis of these tumors by regulating mitosis and key signaling pathways. The distinctive function of NUSAP1 positions it as a central molecule linking mitotic dysregulation with tumorigenesis, exhibiting dual potential as both a diagnostic marker and a therapeutic target. This article primarily reviews the structural characteristics, functional mechanisms, and related signaling pathways of NUSAP1, focusing on exploring the functional mechanisms of NUSAP1 in digestive system neoplasms. The objective is to offer new research perspectives into the diagnosis, treatment, and prognosis evaluation of tumors.

## 1 Introduction

Cancer has long been a huge challenge in both human history and medical science, imposing a heavy burden on global society and economies while posing a significant threat to human health. Despite the continuous advancements in cancer therapeutics, the mortality and incidence rates of cancer are still on the rise. There were approximately 20 million new cancer cases and nearly 10 million cancer-related deaths. Projections indicate that by 2050, the number of new cancer cases will increase by 77% compared to the 2022 figures. Within this group of cancers, the fatality rate for digestive system malignancies is significantly higher than that of other tumor types, and patient outcomes tend to be unfavorable. Epidemiological data reveal that colorectal, gastric, liver, esophageal, and pancreatic cancers are consistently featured among the top ten leading causes of cancer-related deaths (1). Currently, the primary treatments for digestive system tumors encompass surgical resection, radiotherapy, and chemotherapy. Additionally, emerging therapeutic approaches include anti-angiogenic therapy, cancer immunotherapy, and the application of nanotechnology in treatment (2, 3).

The increasing global prevalence of cancer highlights the urgent requirement for focused approaches to cancer prevention and therapy. A major difference between cancer cells and normal cells lies in the uncontrolled proliferation capability of cancer cells, which is driven by dysfunctions in cell cycle regulation, a core mechanism in cancer progression (4). Thus, elucidating the regulatory interactions between the cell cycle and cancer metabolism provides new perspectives on the mechanisms underlying cancer development and establishes a foundation for developing therapeutic approaches that modulate cell cycle regulation in cancer treatment (5). In the context of cell cycle regulatory proteins, a range of therapeutic agents have advanced to clinical application or trial phases, including CDK4/6 inhibitors (6), Aurora-A inhibitors (7), and WEE1 inhibitors (8). Additionally, the roles of increasing cell cycle regulatory proteins in cancer therapy are being continuously uncovered.

Nucleolar and spindle-associated protein 1 (NUSAP1) is a key microtubule-binding protein with critical roles in mitotic regulation. This protein is specifically expressed in dividing cells, where it interacts with microtubules to orchestrate spindle formation, maintain spindle stability, and regulate chromosome dynamics (9–11). NUSAP1 demonstrates dynamic relocalization throughout the cell cycle, with its expression tightly controlled. This regulation is vital for ensuring stable cell division. Consequently, dysregulated NUSAP1 expression levels frequently result in aberrant cell proliferation, leading to embryonic developmental abnormalities and various cancers (12–15). This protein has attracted considerable attention in light of the dynamic cellular processes that NUSAP1 undergoes from spindle assembly to the conclusion of mitosis. Extensive research has demonstrated that NUSAP1 is overexpressed in various malignant tumors such as glioma (16), hepatocellular carcinoma (17), gastric cancer (18), lung cancer (19), prostate cancer (20), breast cancer (21), and bladder cancer (22). This overexpression influences tumor invasion, therapeutic response, and patient prognosis to varying extents.

This study provides a comprehensive analysis of the regulatory features and detailed mechanisms governing NUSAP1 throughout the mitotic cell cycle, along with its influence on the initiation and progression of digestive system cancers, including liver, gastric, esophageal, and pancreatic cancer. Based on these findings, the article analyzes the potential and prospects for therapeutic applications of NUSAP1 in digestive system malignancies.

## 2 NUSAP1 structure and characteristics

### 2.1 The structure of NUSAP1

Nucleolar and spindle-associated protein (NUSAP) is a microtubule- and chromatin-binding protein that plays an essential role in the formation and maintenance of the mitotic spindle (9, 23). Four NUSAP proteins, designated NUSAP1 through NUSAP4, have been identified in *Trypanosoma* *brucei*. Notably, NUSAP1 is a kinetoplastid-specific protein that plays a critical role in the segregation of isochromatids and contributes to the stability of centromere-associated proteins KKIP1 and KKT1. NUSAP2 is a protein in Trypanosoma brucei that contains MAP65/ASE1/PRC1 domains and is involved in facilitating the G2/M phase transition of the cell cycle. NUSAP3 interacts with Kif13–1 and is crucial for promoting chromosome segregation and ensuring the stability of Kif13-1 (24).

**Figure 1:**
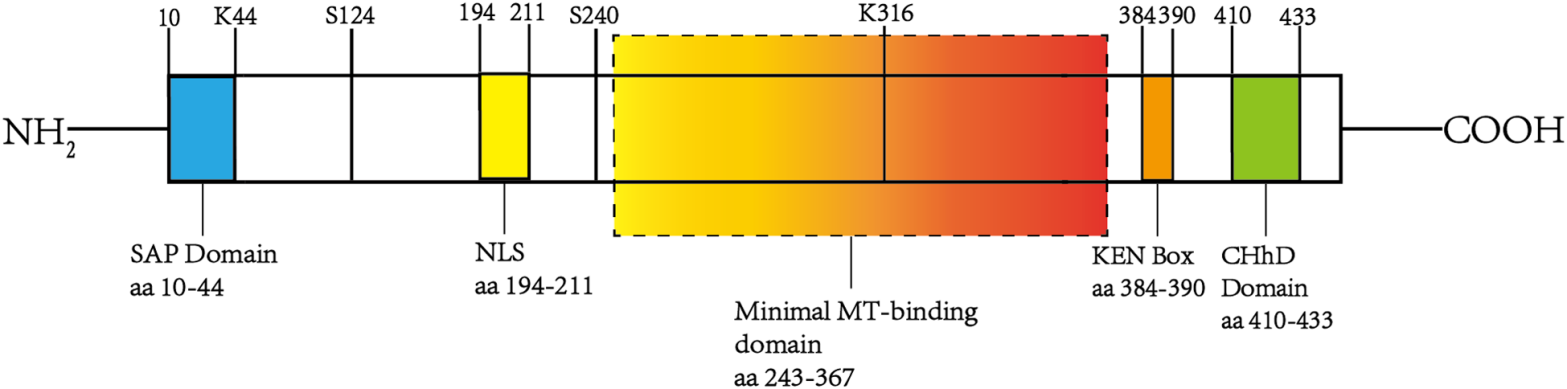
The structure of NUSAP1. The figure illustrates the structural organization and key functional domains of NUSAP1. Specifically, it includes the SAP domain at the NH2 terminus (10–44 aa), the KEN box (384–390 aa), the ChHD domain (410–430 aa), a minimal microtubule-binding region spanning residues 243-367, and a bidirectional nuclear localization signal (NSL) located at 194–211 aa. These domains facilitate interactions with various proteins and signaling molecules, forming the molecular basis for NUSAP1’s diverse functions.

However, the existing literature predominantly focuses on NUSAP1, and this review primarily examines the current understanding of NUSAP1. Human NUSAP1 exhibits significant similarity to its murine counterpart, characterized by selective expression in dividing cells and dynamic localization throughout the cell cycle. The protein has a molecular weight of approximately 55 kDa and includes a single open reading frame spanning 1,281 base pairs. Importantly, its NH2-terminal region contains a putative SAP (SAF-A/B, Acinus, and PIAS) domain (amino acids 10-44), which is believed to function as a DNA-binding site and plays a crucial role in regulating chromosome organization (9, 25, 26). The central domain contains a putative bidirectional nuclear localization signal (194–211 aa), while residues 384–390 form a KEN box motif. The KEN box has been shown to function as a degradation signal for Cdh1 (a WD repeat protein)-mediated anaphase-promoting complex/cyclosome (APC/C) activity, regulating the ubiquitination and degradation of proteins after the completion of the cell cycle, including the APC/C -Cdh1-dependent ubiquitination and degradation of NUSAP (26–28). The COOH-terminal region of NUSAP1 contains a conserved high-charge binding domain (410–430 aa) called the ChHD domain. Similar to other microtubule-associated proteins (MAPs), NUSAP1 interacts with cytoplasmic microtubules via a domain proximal to its COOH terminus. The minimal microtubule-binding domain is between residues 243 and 367 (9, 26) (**Figure 1**).

### 2.2 Characteristics of NUSAP1

Studies have shown that NUSAP1 expression is significantly upregulated during the G2/M phase of the cell cycle, whereas its levels are markedly reduced in the G1 phase. Additionally, NUSAP1 displays dynamic subcellular localization throughout the cell cycle. During interphase, NUSAP1 is primarily found within the nucleus, with a predominant concentration in the nucleolus. As cells enter mitosis, NUSAP1 is released from the nucleus and redistributes to form bundles around the chromosomes. In anaphase, NUSAP1 exhibits strong localization to the central spindle microtubule bundles associated with the chromosomal region. After the completion of mitosis, NUSAP1 is rapidly degraded (9).

Researchers have also observed that overexpression of the NUSAP1 protein results in forming long, curved, and abnormally thick microtubule bundles in the cytoplasm. This accumulation of microtubules leads to significant morphological and viability abnormalities in proliferating cells. Furthermore, inhibition of *NUSAP1* using small interfering RNA (siRNA) induces mitotic delay, causing defects in spindle assembly and cytokinesis. These findings underscore that proper expression and dynamic localization of NUSAP1 are essential for the stable formation of the spindle apparatus and the orderly progression of the mitotic cycle. Both overexpression and knockdown of *NUSAP1* can disrupt normal mitotic processes (26).

## 3 NUSAP1 function and mechanism of action

### 3.1 The function of NUSAP1

In addition to biochemical reconstitution and *in vitro* analyses focused on identifying NUSAP1, earlier research also employed *in vivo* experiments to further elucidate its function in cell proliferation. These studies demonstrated that NUSAP1 deficiency impairs the mitotic process, leading to early embryonic lethality in mice. The underlying mechanisms include the failure of chromosomes to align properly during metaphase at the equatorial plane and separate properly during anaphase due to NUSAP1 deficiency, along with persistent activation of the spindle assembly checkpoint (SAC), ultimately resulting in apoptosis. These findings underscore the critical importance of NUSAP1 in chromatin-induced spindle assembly (12). Furthermore, NUSAP1 also acts as a microtubule-stabilizing factor. It engages with the microtubule depolymerase mitotic centromere-associated kinesin (MCAK), thereby negatively regulating MCAK’s depolymerization activity and enhancing the stability of centromere microtubules. During this interaction, the phosphorylation of MCAK by Aurora B kinase is critical (29). Beyond its direct interaction with microtubules, similar to other MAPs, NUSAP possesses the unique capability to engage with chromatin and accumulate on it. NUSAP can efficiently generate high concentrations of microtubules (MTs) near chromatin or DNA, facilitating the rapid attachment of MTs to chromosomes (10). Additional research has demonstrated that NUSAP1 interacts with the kinesin-like DNA-binding protein Kid during metaphase, enhancing Kid’s association with microtubules and facilitating the generation of polar ejection force (PEF). This interaction regulates chromosome oscillation and ensures proper chromosome alignment (11).

### 3.2 The functional mechanism of NUSAP1

A critical prerequisite for NUSAP1 to exert its function is its translocation from the nucleus to the cytoplasm at the conclusion of interphase, a process that is RanGTP (the small GTPase Ran)-dependent and mediated by RanGTP. RanGTP not only functions as a regulatory switch for the nuclear-cytoplasmic trafficking of multiple spindle assembly factors (SAFs) but also plays a crucial role in driving spindle assembly (30). During interphase, NUSAP1 undergoes differential regulation by three import proteins—Importin(Imp)α, ImpB, and Imp7—to facilitate its nuclear import. Concurrently, the functional activity of NUSAP1 is suppressed, preventing unintended or premature interactions with microtubules and ensuring the proper progression of the mitotic cycle. Subsequently, elevated levels of RanGTP mediate the dissociation of these import proteins from NUSAP1, enabling its translocation to the cytoplasm, where it can interact with microtubules. Notably, the complete release of NUSAP1 from its import proteins requires the concurrent binding of microtubules (10, 13).

### 3.3 Post-translational modifications of NUSAP1

#### 3.3.1 Phosphorylation modification of NUSAP1

During the initiation of mitosis, NUSAP1 undergoes phosphorylation mediated by the Cdk1/cyclin B complex. As a consequence, the interaction between NUSAP1 and microtubules becomes less stable, and this reduced binding persists throughout metaphase until its completion. As the cell cycle progresses into its late phase, NUSAP1 undergoes dephosphorylation and subsequently binds to microtubules, facilitating the formation of the spindle midzone and advancing the cell cycle. In early mitosis, if NUSAP1 remains unphosphorylated, microtubules within the spindle exhibit extensive bundling and form thick, aggregated structures. Consequently, phosphorylation of NUSAP1 during early mitosis is crucial for regulating the proper assembly of the spindle structure (31).

Moreover, RepoMan, a chromosome-associated scaffold protein, can also facilitate the phosphorylation of NUSAP1 at its CDK site, thereby enhancing the activation of NUSAP1 and promoting microtubule assembly (32). Recent studies have highlighted the critical role of NUSAP1 in regulating mitotic spindle assembly. Specifically, NUSAP1 facilitates the cross-linking of microtubules by the sliding motor protein Eg5, mediating the sliding of antiparallel interpolar microtubules and thereby extending the length of the metaphase spindle. Additionally, Aurora A phosphorylates NUSAP1 at Ser-240, enhancing its interaction with the microtubule depolymerase Kif2A on the spindle. This interaction reduces the concentration of Kif2A at the spindle poles, thereby inhibiting microtubule depolymerization and ensuring the stability of the mitotic spindle (33).

#### 3.3.2 SUMO modification of NUSAP1

SUMOylation also influences NUSAP1 activity. Notably, Ran-binding protein 2 (RANBP2), a nuclear pore protein with SUMO1 E3 ligase activity, is essential for the mitotic SUMO pathway (34, 35). The interaction between NUSAP1 and RANBP2 stabilizes NUSAP1 at the ends of microtubules. Depletion of RANBP2 results in a marked decrease in NUSAP1 levels. Moreover, NUSAP1 forms SUMO2/3-conjugated products at K44 and K316 residues in a RANBP2-dependent manner. These sites are situated within the SAP domain and microtubule-binding domain, respectively. Research findings indicate that mutation at K44 impairs NUSAP1’s binding to chromatin, thereby disrupting chromosome alignment, while mutation at K316 results in microtubule depolymerization and accelerates mitotic spindle disassembly (36).

#### 3.3.3 Ubiquitination modification of NUSAP1

**Figure 2:**
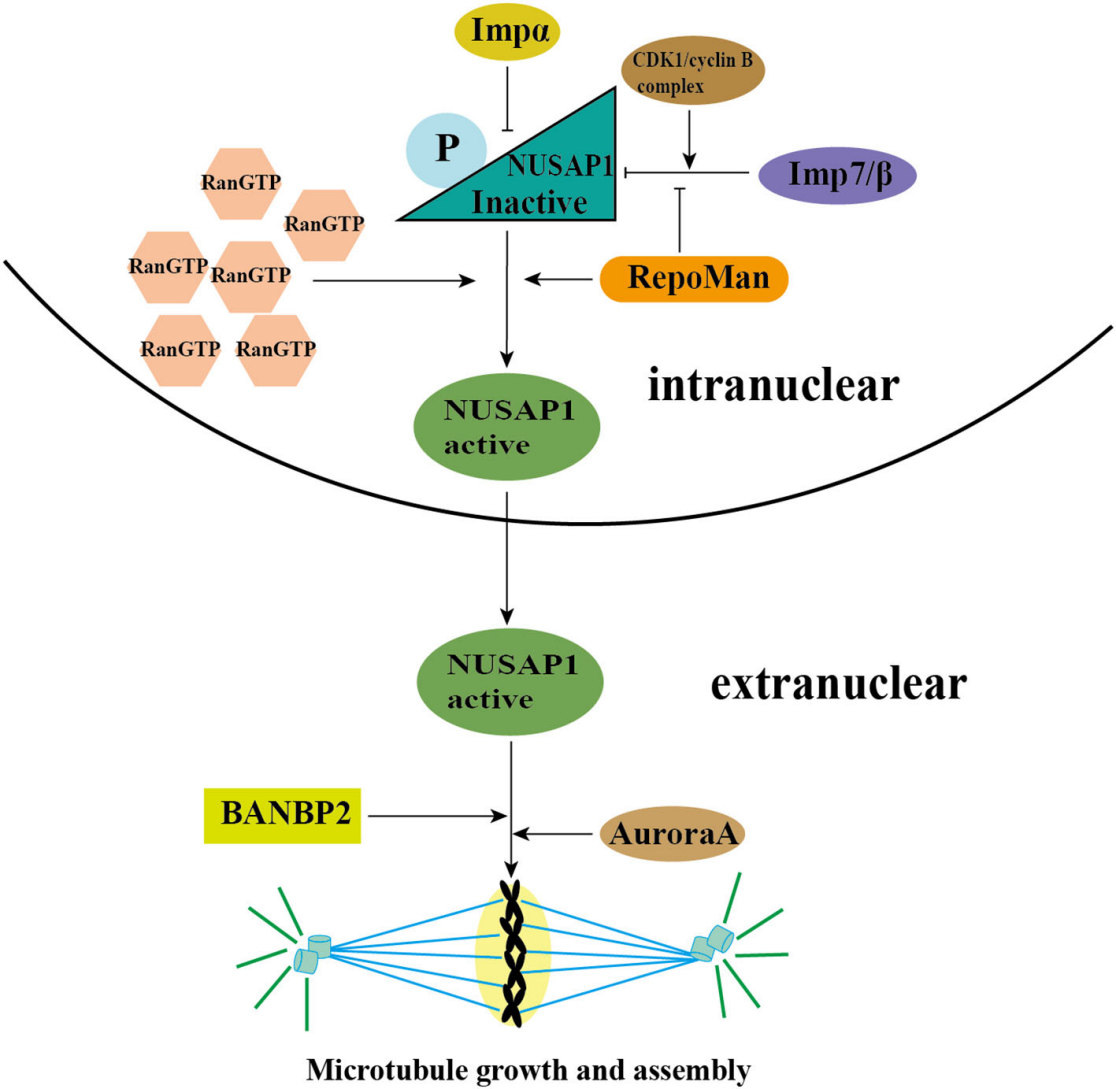
The activation of NUSAP1 and MT assembly. During interphase, NUSAP1 is differentially regulated by three importins—Importin (Imp) α, ImpB, and Imp7—to facilitate its nuclear localization. Additionally, NUSAP1 is phosphorylated by the CDK1/cyclin B complex, which inhibits its function and ensures proper progression through the mitotic cycle. Subsequently, high concentrations of RanGTP mediate the dissociation of these importins from NUSAP1, allowing it to translocate to the cytoplasm and interact with microtubules. RepoMan promotes this activation process by phosphorylating the CDK site of NUSAP1, thereby facilitating its separation from the imports. Furthermore, NUSAP1 is phosphorylated at Ser-240 by Aurora A kinase, which inhibits microtubule depolymerization and ensures the stability of the mitotic spindle. RANBP2, which possesses SUMO1 E3 ligase activity, interacts with NUSAP1 and stabilizes it at microtubule ends. Moreover, NUSAP1 forms SUMO2/3 conjugates at lysine residues K44 and K316 in a RANBP2-dependent manner. These modifications occur within the SAP domain and microtubule-binding domain, respectively, and are crucial for the proper formation and maintenance of the spindle structure.

NUSAP1 expression is rigorously regulated during the entire cell cycle to guarantee accurate mitotic progression. Furthermore, NUSAP1 degradation occurs via ubiquitination (37). In the G1 phase, NUSAP1 undergoes ubiquitination and subsequent degradation mediated by the APC/C-Cdh1 E3 ligase. The KEN box within NUSAP1 acts as the specific recognition element for APC/C-Cdh1 (26, 27) (**Figure 2** and **Table 1**).

**Table 1:** Post-translational modifications of NUSAP1.

Types of post-translational modifications	The relevant enzymes or proteins	Modification site	The result of the effect	References
Phosphorylation	The Cdk1/cyclin B complex	——	NUSAP1 remains in its inactive state.	(31)
	RepoMan	CDK	NUSAP1 activation and MT assembly	(32)
	AuroraA	Ser-240	Inhibit microtubule depolymerization to ensure the stability of the spindle.	(33, 33)
	ATM(phosphoinositide 3-kinase family)	Ser124	Ectopic expression of NUSAP results in mitotic arrest.	(151)
SUMOylation	RANBP2	K44	Promoting NUSAP1 binding to chromatin ensures precise chromosome alignment.	(34–36)
		K316	Inhibit microtubule depolymerization and promote spindle stability.	(34–36)
Ubiquitination	The APC/C-Cdh1 E3 ligase	KEN box	NUSAP1 ubiquitination and subsequent degradation ensure the normal progression of the mitotic cycle.	(26, 27)

## 4 NUSAP1 and signaling pathways

Recent studies have demonstrated that NUSAP1 plays a pivotal role in cell division and interacts with multiple signaling pathways, thereby contributing to tumorigenesis and progression. Accumulating evidence suggests that NUSAP1 is closely associated with key biological processes such as DNA repair, cellular metabolism, and cell cycle regulation (38–40). Therefore, an in-depth exploration of the interaction mechanisms between NUSAP1 and these signaling pathways will contribute to the formulation of innovative anti-tumor therapeutic approaches (**Table 2**).

**Table 2:** Major signaling pathways involving NUSAP1 and its role in tumors.

Signaling pathway	Mechanism of function	Tumor categories	References
Wnt/B-catenin signaling pathway	Promotes the nuclear translocation of B-catenin and enhances its transcriptional activity.	Breast cancer, Cervical carcinoma	(21, 44)
Hedgehog signaling pathway	Promotion of GLI1 nuclear translocation facilitates its accumulation within the nucleus.	Astrocytoma, Basal cell carcinoma	(59, 60)
PI3K/Akt signaling pathway	Regulates phosphorylation of PI3K and Akt.	Lung cancer, Nephroblastoma	(65, 68, 70)
TGF-B signaling pathway	The enhancement of EMT is promoted through the regulation of TGFBR1 and Smad2/3 expression.	Bladder cancer, Gastric cancer	(76–78)
SHCBP1/JAK2/STAT3 signaling pathway	NUSAP1 inhibits JAK2/STAT3 phosphorylation through SHCBP1, affecting the immune microenvironment.	Hepatocellular carcinoma	(105)
Hippo signaling pathway	NUSAP1 enhances the stability of YAP1, promoting its nuclear translocation and transcriptional activity.	Gastric cancer	(18)
AMPK/PPARγ signaling pathway	NUSAP1 regulates cellular metabolism and energy homeostasis.	Breast cancer	(39)
DNA damage repair pathway	NUSAP1 promotes the SUMOylation of RAD51 to prevent its degradation.	Chronic lymphocytic leukemia	(38)

### 4.1 NUSAP1 and Wnt/B-catenin signaling pathway

The Wnt/B-catenin signaling pathway is a highly conserved molecular mechanism widely involved in biological processes such as embryonic development, cell proliferation, and differentiation (41). When this signaling pathway is dysregulated and aberrantly activated, it can contribute to carcinogenesis and is closely associated with tumor malignancy and poor prognosis (42, 43). Activation of the Wnt/B-catenin signaling pathway initiates with Wnt ligands binding to its frizzled receptors and lipoprotein receptor-related protein-5 or -6 (LRP5/6) co-receptors, which subsequently triggers B-catenin translocation and nuclear accumulation. Next, B-catenin forms a complex cell nucleus transcription factor with T-cell factor/lymphoid enhancer factor (TCF/LEF), activating target gene transcription (44).

The Wnt/B-catenin signaling pathway exerts a crucial influence on cancer metastasis, encompassing key processes such as the epithelial-mesenchyme transition (EMT) and the modulation of cancer stem cell (CSC) properties (44). Accumulating evidence demonstrates that NUSAP1 promotes cancer cell proliferation and invasion by stimulating both Wnt/B-catenin signaling and EMT pathways (21). As an important post-translational modification, SUMOylation is involved in regulating oncogenic processes, the cell cycle, and apoptosis (45). T-cell factor (TCF) serves as a critical transcription factor in the Wnt signaling pathway and plays an essential role in establishing the positive feedback loop of this pathway (46). Upon Wnt protein stimulation in the extracellular environment, B-catenin translocates to the nucleus and transforms TCF from a transcriptional repressor into a transcriptional activator (47–49). Research has indicated that highly expressed NUSAP1 induces the nuclear translocation of B-catenin, significantly enhances the transcriptional activity of B-catenin/TCF, and simultaneously promotes the SUMOylation of TCF4. By increasing nuclear B-catenin levels, NUSAP1 initiates the activation of the Wnt/B-catenin signaling pathway, which consequently supports the metastasis of cancer cells. Furthermore, the upregulation of NUSAP1, through its interaction with SUMO E3 ligase RanBP2, further induces the activation of the Wnt/B-catenin signaling pathway, promoting CSC properties and the EMT process (44).

Meanwhile, as the central mediator of Wnt/B-catenin signaling, B-catenin is subject to stringent regulation of its cellular concentration (50). When Wnt signaling is inactive, the concentration of B-catenin is minimal due to ongoing degradation facilitated by the destruction complex (51). Glycogen synthase kinase-3B (GSK-3B), as one of the components of the destruction complex, promotes the phosphorylation of B-catenin, leading to its ubiquitination and subsequent degradation (51–53). Research has shown that in nasopharyngeal carcinoma (NPC), the overexpression of *NUSAP1* leads to increased phosphorylation of GSK-3B, thereby reducing its enzymatic activity. Consequently, this reduction in GSK-3B activity decreases the phosphorylation of B-catenin, ultimately enhancing the activation of the Wnt/B-catenin signaling pathway. Moreover, inhibition of GSK-3B not only improves the activity of the Wnt/B-catenin signaling pathway but also restores the suppression of signaling caused by *NUSAP1* knockout. These findings indicate that NUSAP1 plays a critical role in the activation of the Wnt/B-catenin signaling pathway, with its function being mediated through GSK-3B (54). However, whether GSK-3B inhibition potentiates NUSAP1 activity in digestive system cancers remains experimentally unexplored.

While current studies have unequivocally shown that NUSAP1 positively modulates the Wnt/B-catenin pathway through multiple mechanisms, including B-catenin nuclear translocation, SUMOylation of TCF4, and inhibition of GSK-3B phosphorylation, it remains to be elucidated whether Wnt signaling feedback regulates NUSAP1 expression through the classical ligand-receptor cascade. The underlying regulatory interaction network between these two factors still requires systematic investigation.

The aberrant activation of the Wnt/B-catenin signaling pathway plays a pivotal role in tumorigenesis and tumor progression, with its regulatory mechanisms encompassing molecular interactions at multiple levels. The transcription factor myocyte enhancer factor 2D (MEF2D) is primarily localized in the nucleus and regulates cellular processes, including growth, differentiation, survival, and apoptosis (55). MEF2D directly binds to the *NUSAP1* promoter, thereby enhancing the transcription of NUSAP1 mRNA and subsequent protein expression. Notably, the downregulation of MEF2D inhibits B-catenin accumulation and nuclear translocation, consequently diminishing Wnt/B-catenin signaling activity in cancer cells. However, *NUSAP*1 overexpression can reverse the inhibitory effect of MEF2D knockdown on the activation of the Wnt/B-catenin signaling pathway. The interaction between NUSAP1 and MEF2D enhances the activity of the Wnt/B-catenin signaling pathway, promoting tumor progression (56). Similarly, increased expression of ankyrin repeat domain 22 (ANKRD22) was observed to markedly elevate the levels of NUSAP1 expression. Additionally, elevated ANKRD22 levels led to greater nuclear accumulation of B-catenin, which in turn triggered activation of the Wnt/B-catenin signaling pathway. Further studies have demonstrated that ANKRD22 indirectly activates the Wnt/B-catenin signaling pathway by modulating the expression of NUSAP1, consequently facilitating the malignant progression of cancer cells (57). These results suggest that a multitude of upstream factors, such as MEF2D and ANKRD22, regulate the Wnt/B-catenin signaling pathway activity by modulating NUSAP1 expression, which in turn has a crucial role in the progression of tumors.

### 4.2 NUSAP1 and hedgehog signaling pathway

The Hedgehog (HH) signaling pathway, first discovered through genetic studies in *Drosophila melanogaster*, is essential for governing the early stages of embryonic development (58). The *glioma-associated oncogene (GLI)* protein is one of the major signal transducers of the HH signaling pathway. The overexpression of NUSAP1 leads to the activation of the HH signaling pathway. Specifically, increased NUSAP1 expression induces the translocation of GLI1 from the cytoplasm to the nucleus, leading to nuclear accumulation of GLI1 and subsequent stimulation of the HH signaling pathway. This leads to an increase in the expression of downstream target genes. This process enhances the invasiveness of tumor cells and facilitates the growth and metastasis of astrocytoma (59). Besides, NUSAP1 significantly influences the proliferation, migration, invasion, and DNA damage in basal cell carcinoma. These effects may be facilitated by activating the HH signaling pathway (60).

### 4.3 NUSAP1 and PI3K/Akt signaling pathway

The PI3K/Akt signaling pathway is essential in regulating the cell cycle and is intimately associated with cell quiescence, proliferation, and cancer development. Abnormal activation of its signaling transduction has oncogenic effects (61, 62). *B-cell translocation gene 2 (BTG2)* is a member of the antiproliferative gene family and is involved in regulating the cell cycle and apoptosis while also exerting tumor-suppressive functions (63, 64). The knockdown of *NUSAP1* results in upregulated *BTG2* expression and concurrent inhibition of PI3K and Akt phosphorylation, thereby reducing the activity of the PI3K/Akt signaling pathway. This suggests that NUSAP1 may activate the PI3K/Akt signaling pathway by suppressing BTG2 (65). The *homeobox (HOX)* genes encode a family of pivotal transcription factors for regulating cell differentiation and development (66). One member of this family, the homeobox protein Hox-B2 (HOXB2), has been found to enhance the malignant behavior of malignant cells, thus playing an essential role in cancer progression (67). Investigations have revealed that HOXB2 significantly enhances *NUSAP1* expression and can restore the inhibited PI3K/Akt signaling pathway following NUSAP1 silencing. This suggests that HOXB2 may upregulate NUSAP1 expression through activation of the PI3K/Akt signaling pathway, consequently promoting cancer cell proliferation, invasion, and migration (68). The *mammalian target of rapamycin (mTOR)* serves as a crucial downstream effector of the PI3K/Akt signaling pathway (69). Research has shown that NUSAP1 knockdown substantially reduces the phosphorylation levels of both AKT and mTOR, along with an increase in apoptosis rates. These findings indicate that NUSAP1 promotes cancer development by activating the PI3K/Akt/mTOR signaling pathway (70). Elevated *PI3K* activity strongly correlates with cellular malignant transformation and tumorigenesis (71). The most direct therapeutic approach to inhibiting the PI3K/Akt signaling pathway is to target *PI3K* itself (72). To date, multiple PI3K inhibitors (e.g., BKM120) with potential anticancer applications have been synthesized (73). NUSAP1 may affect cell growth and survival through the regulation of cell cycle progression and apoptosis-related pathways. Targeting *NUSAP1* may significantly enhance the therapeutic efficacy of existing PI3K inhibitors, but its clinical potential necessitates further experimental validation and comprehensive clinical investigation.

### 4.4 NUSAP1 and TGF-B signaling pathway

Transforming growth factor-B (TGF-B) is a highly conserved multifunctional cytokine involved in modulating diverse signaling pathways throughout both embryonic and adult stages. It performs critical regulatory functions in cell differentiation, proliferation, and cell- or tissue-specific motility (74). The TGF-B signaling pathway exerts a dynamic influence on human cancer progression, demonstrating inhibitory effects during initial cancer development but facilitating cancer advancement in later stages (75). The TGF-B signaling pathway drives the proliferation and metastasis of cancer cells by inducing the EMT process. TGF-B receptor type 1 (TGFBR1) is crucial in this process (76, 77). The experiment shows that the expression level of NUSAP1 is positively correlated with TGFBR1 and the downstream effector factors Smad2/3 of the TGF-B signaling pathway. Overexpression of NUSAP1 not only up-regulates the expression levels of TGFBR1 and Smad2/3 but also promotes the expression of mesenchymal cell markers, such as Vimentin and N-cadherin, while simultaneously inhibiting the expression of the epithelial cell marker E-cadherin (78, 79). Inhibiting TGFBR1 expression in NUSAP1-overexpressing cells significantly suppresses their invasive and metastatic capabilities while also markedly decreasing the expression levels of p-Smad2/3 and EMT-related proteins. The research results suggest that *NUSAP1* may activate the TGF-B signaling pathway by directly up-regulating TGFBR1, thereby mediating the EMT process and promoting tumor cell proliferation, migration, and invasion (78).

### 4.5 The Hub Role of NUSAP1 in Signaling Pathways

As a critical microtubule-associated protein and cell cycle regulator, NUSAP1 exerts central control over tumorigenesis and progression by modulating multiple signaling pathways through its intricate molecular mechanisms. Existing research evidence demonstrates that NUSAP1 exerts multi-level regulation on signaling pathways, including Wnt/B-catenin, Hedgehog, PI3K/Akt, and TGF-B, thereby establishing a precise regulatory network.

NUSAP1 achieves multifunctional regulation via its unique structural domains, including chromatin binding, microtubule stabilization, and protein stability modulation (10, 29, 36). Notably, the regulatory function of NUSAP1 is distinguished by its significant multi-pathway synergistic characteristics. Within the TGF-B signaling pathway, NUSAP1 upregulates TGFBR1 expression, leading to enhanced Smad2/3 phosphorylation and consequently promoting the EMT process (78). Another study demonstrated that transmembrane protein 64 (TMEM64) activates the Wnt/B-catenin signaling pathway by facilitating B-catenin nuclear translocation. Additionally, in cells with TMEM64 knockdown, the expression levels of mesenchymal markers Vimentin and N-cadherin were significantly decreased, whereas the expression of the epithelial marker E-cadherin was markedly upregulated (80). These findings suggest that EMT may serve as a common downstream effector of the Wnt/B-catenin and TGF-B signaling pathways. Moreover, NUSAP1 is likely to synergistically promote EMT via both pathways, thereby enhancing tumor cell proliferation, migration, and invasion. Given the central role of NUSAP1 in pro-cancer signaling pathways, targeting *NUSAP1* for inhibition may enable coordinated blockage of multiple pathways, thereby establishing it as a highly promising therapeutic target for cancer treatment.

## 5 NUSAP1 and digestive system neoplasms

### 5.1 NUSAP1 and liver cancer

#### 5.1.1 Overview of liver cancer

Liver cancer ranks as the sixth most common malignancy worldwide and is geographically widespread. It is the third leading cause of cancer-related mortality, following lung and colorectal cancers (1). Liver cancer can be categorized into multiple subtypes. Among these, hepatocellular carcinoma (HCC) constitutes approximately 80% of primary liver cancers, while intrahepatic cholangiocarcinoma (iCCA) accounts for about 15% (81). Over the past few decades, the global advancement in medical and healthcare standards has led to a decline in both the incidence and mortality rates associated with liver cancer. Nevertheless, the five-year survival rate remains suboptimal, as most patients are diagnosed with HCC at an advanced stage, resulting in a poor prognosis (82). Current treatment modalities for liver cancer primarily encompass surgical excision, transcatheter arterial chemoembolization (TACE), radiofrequency ablation, pharmacological interventions such as tyrosine kinase inhibitors (TKIs), and immunotherapy (83). In light of the availability of diverse treatment options, the complexity of liver cancer development and its high post-treatment recurrence rate underscores the urgent necessity to identify effective biomarkers and therapeutic targets and elucidate their relationship with changes in the tumor microenvironment in HCC management. NUSAP1, serving as a key regulator of the cell cycle, plays a pivotal role in tumor progression and alterations within the tumor microenvironment (15). Moreover, the potential link between this factor and HCC has attracted considerable attention from a wide range of researchers.

It is commonly acknowledged that the development of HCC is not attributable to a single gene but rather emerges from the interplay of multiple genetic factors and environmental influences. Leveraging advanced bioinformatics tools, numerous studies have identified key hub genes with essential pathological roles in liver cancer progression by employing various approaches, including database mining, gene network interaction analysis, survival analysis, and risk evaluation. In 2020, a study aimed to identify potential therapeutic target genes for HCC and ultimately pinpointed 10 hub genes. Among these, six genes—*OIP5*, *ASPM*, *NUSAP1*, *UBE2C*, *CCNA2*, and *KIF20A*—were recognized as novel hub genes in the context of HCC (84). A separate study identified six genes—*CDKN3*, *ZWINT*, *KIF20A*, *NUSAP1*, *HMMR*, and *DLGAP5*—that are closely associated with HCC prognosis through COX proportional hazards regression analysis and the development of a prognostic model (85). Both studies performed survival analysis on NUSAP1 and consistently concluded that its overexpression is significantly associated with reduced survival time in HCC patients. Furthermore, *NUSAP1* is one of the hub genes associated with immune infiltration and can predict poor prognosis in HCC patients (86, 87).

As early as 2013, studies reported that NUSAP1 expression levels in HCC tissues were markedly higher than in normal tissues. Moreover, elevated NuSAP1 protein levels were significantly correlated with early postoperative recurrence in HCC patients (88). A study utilized RT-qPCR and western blotting techniques to examine the expression levels of *NUSAP1* in 47 paired tumor and adjacent non-tumor tissues, demonstrating that both mRNA and protein levels of *NUSAP1* were significantly upregulated in tumor tissues. Furthermore, lentivirus-mediated downregulation of *NUSAP1* resulted in a significant reduction in cell proliferation and invasion (89). In recent years, there has been a rise in research efforts focused on uncovering the precise mechanisms through which NUSAP1 influences HCC progression. Several studies have demonstrated that NUSAP1 accelerates HCC proliferation by regulating the G1-to-S phase transition (90, 91). Furthermore, NUSAP1 can influence CD4+ T cell resting memory and M0 macrophages via potential mechanisms. Additionally, lower expression levels of *NUSAP1* have been linked to enhanced immunotherapy outcomes in HCC patients. This indicates that NUSAP1 could serve as a promising therapeutic target for immunotherapy in HCC (91). *NUSAP1* has been recognized as a specific gene involved in the progression from non-alcoholic fatty liver disease (NAFLD) (92), HBV infection (93, 94), and liver cirrhosis (95, 96) to HCC. Consequently, NUSAP1 is likely to play a critical role in the carcinogenesis of liver diseases. Inhibiting NUSAP1 expression may hinder or delay the progression of various chronic liver conditions to HCC, thereby highlighting its substantial potential as a therapeutic target for HCC prevention.

#### 5.1.2 The mechanism by which NUSAP1 regulates HCC

Beyond bioinformatics analysis, recent studies have also concentrated on molecular biology experiments to elucidate the specific molecular mechanisms by which NUSAP1 regulates HCC. Researchers developed three HCC mouse models with varying pathogenicities and conducted a combined analysis of miRNA and mRNA to identify a previously unrecognized miRNA, miR-193a-5p. Using conventional bioinformatics target prediction and comprehensive mRNA transcriptome analysis, they established that *NUSAP1* is a critical target of miR-193a-5p. During this investigation, it was observed that miR-193a-5p levels were reduced in both mouse and human HCC cells and tissues, leading to increased *NUSAP1* expression. NUSAP1 regulates the cell cycle, promoting HCC cell proliferation, survival, and metastatic potential, thereby reducing patient survival time. These findings indicate that NUSAP1 serves as a key mediator in the miR-193a-5p-regulated progression of HCC. Upregulating miR-193a-5p expression or inhibiting *NUSAP1* to disrupt the miR-193a-5p/NUSAP1 axis may represent a promising therapeutic strategy for HCC (97).

In addition to miR-193a-5p, further studies have revealed that NUSAP1 functions as a critical target for miR-122 in the progression of HCC, regulating cell cycle-related processes. In the absence of miR-122 (i.e., under knockout conditions), a significant elevation in *NUSAP1* expression was observed (98). In the context of HBV infection, the HBV X protein (HBx) downregulates miR-18b by inducing methylation of the CpG island in the miR-18b gene promoter. Using miRNA target gene prediction analysis, researchers identified *NUSAP1* as a potential target of miR-18b and noted that the downregulation of miR-18b led to increased NUSAP1 expression. Follow-up studies revealed that HBx-induced upregulation of *NUSAP1* significantly enhanced liver cancer cell proliferation in both experimental and physiological settings, thereby promoting hepatocarcinogenesis. These results highlight a potential pathway linking HBV infection to liver cancer development (99). Circular RNA Hsa_circ_0002124 originates from intron 9 of the *NUSAP1* gene and exhibits significantly increased expression levels in HCC tissues. Subsequent studies have demonstrated that hsa_circ_0002124 promotes HCC cell proliferation by modulating the expression of key proteins associated with the MAPK signaling pathway in HCC cells (100).

Besides modulating the cell cycle to promote the proliferation of liver cancer cells, NUSAP1 can also enhance the cancer stemness characteristics of HCC cells and increase the early recurrence of HCC. Cancer stem cells, also known as tumor-initiating cells, are the major factors causing the difficulty in treating liver cancer and the high recurrence rate (101, 102). Based on the analysis of existing public datasets and HCC patient cohort data, it was observed that NUSAP1 expression is markedly upregulated in liver tumors and is strongly associated with early recurrence in HCC. Further studies using multiple mouse models revealed that NUSAP1 facilitates the activation of the STAT3 signaling pathway by interacting with receptors for activated C kinase 1 (RACK1), thereby promoting stem cell-like characteristics in HCC cells and contributing to early recurrence. These findings suggest that NUSAP1 may serve as a valuable predictive marker for postoperative intervention in HCC patients and a sensitive indicator of early recurrence (17).

The E2F8 transcription factor, a member of the E2F family, has been shown to exhibit significantly elevated expression levels in HCC and harbors the potential to promote cellular proliferation (103). Recent studies have uncovered an interaction between NUSAP1 and E2F8, wherein *NUSAP1* serves as a downstream target of E2F8. E2F8 enhances cisplatin resistance in liver cancer cells by activating *NUSAP1*, which in turn inhibits DNA damage. Conversely, silencing *NUSAP1* results in cell cycle arrest in liver cancer cells increases DNA damage, and sensitizes these cells to cisplatin-based chemotherapy (104). These findings suggest that the E2F8/NUSAP1 axis may serve as a potential target for mitigating cisplatin resistance in HCC and offer a novel strategy to improve chemosensitivity in liver cancer. NUSAP1 exerts a potential influence on modulating the tumor immune microenvironment in HCC. Specifically, NUSAP1 interacts with SHC and spindle-associated protein 1 (SHCBP1) to inhibit the phosphorylation of the Janus kinase 2/signal transducer and activator of transcription 3 (JAK2/STAT3) pathway. The activation of the SHCBP1/JAK2/STAT3 pathway can suppress the differentiation of peripheral blood mononuclear cells (PBMCs) into dendritic cells (DCs), leading to tumor immune evasion. Consequently, NUSAP1 may represent a viable target for HCC immunotherapy, promoting both HCC cell apoptosis and DC generation (105).

### 5.2 NUSAP1 and gastrointestinal neoplasms

#### 5.2.1 NUSAP1 and esophageal cancer

Globally, esophageal cancer (EC) ranks eleventh in terms of cancer incidence and is responsible for the 7th highest number of cancer-related deaths worldwide (1). Additionally, it is also listed as the 7th most frequently occurring cancer in China (106). EC comprises two major histological subtypes: esophageal squamous cell carcinoma (ESCC) and esophageal adenocarcinoma (EAC). Both subtypes are characterized by unique epidemiological and clinical characteristics (107). EC currently confronts serious challenges in clinical practice, including extensive treatment requirements, severely limited health-related quality of life (HRQOL), and poor prognosis (108). At present, most treatment methods, chemotherapy or chemoradiotherapy, followed by extensive surgery (108), but these often lead to adverse complications and a decline in quality of life. Therefore, elucidating the underlying molecular mechanisms related to the progression of EC is one of the effective approaches to improving existing treatment methods and poor prognosis. Research has shown that *NUSAP1* is an independent predictor of ESCC, and its expression is significantly correlated with the malignancy and invasive characteristics of ESCC. Patients with lower NUSAP1 expression levels demonstrate extended overall survival (OS), reduced tumor proliferation, and a more favorable clinical prognosis (109). Esophageal chemical burns may be one of the potential carcinogenic factors for EC. Simulation analyses suggest that *NUSAP1* could be considered a key shared gene between chemical burns and EC (110). These researches indicate that NUSAP1 may play a decisive function in the occurrence and development of EC by modulating cancer cell proliferation and apoptosis.

#### 5.2.2 NUSAP1 and gastric cancer

Gastric cancer (GC) is ranked fifth worldwide in both incidence and mortality rates (1), and it is also the fifth most frequently diagnosed cancer in China (106). GC often exhibits an insidious onset, with no symptoms or nonspecific symptoms in the early stages, making diagnosis difficult (111). Most patients are diagnosed at an advanced stage of GC, resulting in a high mortality rate (112). In 2022, the global mortality from GC was estimated to be approximately 660,000 (1). Most current clinical treatments for GC are still traditional surgery combined with radiotherapy and chemotherapy. This approach not only impairs the growth, development, and differentiation of normal cells, leading to a decline in immune function, but also has the potential to induce severe side effects (113). In recent years, advancements in molecular biology and genomics have led to a growing focus on identifying novel molecular targets and biomarkers for GC, thereby offering the potential to improve the early diagnosis rate and treatment efficacy.

NUSAP1 exerts a significant influence on the development and clinical treatment of GC through its involvement in regulating several critical signaling pathways. The signaling pathway governs cellular processes such as proliferation, differentiation, and survival in multicellular animals (114, 115). Within this pathway, Yes-associated protein 1 (YAP1) serves as the core effector molecule and plays a critical role in the progression of various human cancers, including GC (18). Research demonstrates a direct interaction between NUSAP1 and YAP1, with the expression level of NUSAP1 positively correlated to the stability of YAP1. This implies that NUSAP1 may enhance the transcriptional activity of YAP1 by stabilizing it, thereby coordinating the Hippo signaling pathway and promoting the malignant behavior of GC cells. These findings highlight the critical role of NUSAP1 in GC progression (18). Previous studies have demonstrated that the Hippo-YAP1 signaling pathway plays a pivotal role in gastric tumorigenesis, and maintaining the stability of YAP1 protein expression represents one of the core mechanisms enabling its diverse functional activities (116). NUSAP1 exhibits potential as a therapeutic target for YAP1-driven tumors by stabilizing YAP1 and co-regulating critical oncogenic pathways. Future studies should focus on elucidating the underlying molecular mechanisms and investigating specific inhibition strategies, thereby offering novel insights into combined treatment approaches for GC.

Moreover, mTOR has been shown to impact tumor progression through dysregulated cellular signaling pathways in multiple cancers. Its signal is mediated by two distinct complexes, mTOR complex 1 (mTORC1) and 2 (mTORC2). Previous studies have established that the mTORC1 signaling pathway critically regulates cellular processes, including the cell cycle, growth, apoptosis, EMT, migration, and invasion across diverse malignancies. Recent research has further revealed that downregulation of NUSAP1 can inhibit the mTORC1 signaling pathway, thereby suppressing the proliferation, migration, and invasion of GC cells (117–119).

The combined application of anticancer bioactive peptides (ACBP) and oxaliplatin (OXA) effectively inhibits the proliferation of the GC cell line MKN-45 and induces its apoptosis. This process is accompanied by the decreased expression of multiple proteins, such as NUSAP1, indicating that the downregulation of NUSAP1 may suppress GC cell proliferation and enhance the anti-tumor efficacy of medicines (120). In addition, NUSAP1 enhances cellular radioresistance by inhibiting the ubiquitination of ANXA2. Meanwhile, miR-129-5p directly targets *NUSAP1* to reduce its expression, thereby diminishing the radioresistance of GC cells. This suggests that modulating the miR-129-5p/NUSAP1/ANXA2 pathway may provide a novel strategy to improve the efficacy of radiotherapy for GC (121). Another study has reported a positive correlation between the expression level of NUSAP1 and the risk of peritoneal metastasis in patients with GC, highlighting its potential value as a biomarker (122).

#### 5.2.3 NUSAP1 and colorectal cancer

Colorectal cancer (CRC) is a prevalent and fatal malignancy, with the third highest incidence rate and second highest mortality rate in the world. CRC is the second most frequently diagnosed in China (1, 106). CRC tends to show obvious symptoms only in the late stages. Timely screening and personalized therapy are the keys to improving patients’ prognosis and survival rates. With a deepening understanding of CRC, future treatment strategies will become more diverse and effective (123).

Several studies have confirmed the presence of multiple differentially expressed genes (DEGs), including *NUSAP1*, in CRC tissues. These results consistently suggest that the upregulation of *NUSAP1* is potentially associated with poor prognosis in CRC patients (124–127). *NUSAP1* and its interacting miRNAs affect CRC growth and hold potential as biomarkers for prognosis in CRC patients (126). It was also found that *NUSAP1* expression is significantly upregulated in CRC tissues and cell lines. Knockdown of *NUSAP1* to silence its expression effectively inhibits cancer cells’ proliferation, invasion, migration, and EMT. Elevated DNA methyltransferase 1 (DNMT1) expression promotes CRC proliferation, metastasis, and invasion. In contrast, silencing *NUSAP1* markedly decreases the expression of *DNMT1* at both mRNA and protein levels, indicating that NUSAP1 may affect the malignant behavior of cancer cells through modulation of DNMT1 expression (128). Further study has demonstrated that NUSAP1 expression in CRC tissues is markedly elevated compared to normal tissues. NUSAP1 expression is correlated with histopathological grading, depth of invasion, lymph node metastasis, and TNM stage. In patients with advanced-stage CRC, high NUSAP1 expression is significantly associated with poor prognosis (129). These findings suggest that the expression level of NUSAP1 relates to the prognosis of CRC, implying its potential as a prognostic marker and therapeutic target.

### 5.3 NUSAP1 and pancreatic cancer

Pancreatic cancer (PC) is among the malignancies with the poorest prognosis. In 2022, there were 511,000 new cases of PC globally, leading to 467,000 deaths. This accounted for approximately 5% of all global cancer deaths, making PC the sixth leading cause of cancer-related mortality (1). PC is the 10th most common cancer in China (106). Despite recent advances in PC diagnostics and treatments, the five-year survival rate for patients remains extremely low, at approximately 4% (130). The molecular biological mechanisms underlying the occurrence and development of PC are complex and diverse and require further study. In recent years, numerous data analyses and experimental studies have demonstrated that NUSAP1 expression in PC tissues is higher than in normal pancreatic tissues. Its high expression is closely associated with OS and disease-free survival (DFS) in PC patients, suggesting that NUSAP1 may play a pivotal and indispensable role in the proliferation, migration, and invasion of PC cells and could function as a prognostic indicator for PC patients (131–134).

NUSAP1 is involved in regulating the process of PC occurrence, development, and metastasis through multiple molecular mechanisms. Research has established that methionine stress regulates the cell cycle and mitosis of PC cells. Under methionine stress conditions, NUSAP1 expression is significantly downregulated in the PC cell lines, coinciding with observed cell cycle arrest and mitotic abnormalities. This indicates that NUSAP1 could be essential for the proliferation and division of PC cells. This indicates that NUSAP1 could be necessary for PC cell proliferation and division processes (135). Altered NUSAP1 expression has been hypothesized to be associated with cancer cell growth and proliferation. Under hypoxic conditions, NUSAP1 expression in PC cells increases more than tenfold, suggesting that NUSAP1 may facilitate cancer cell proliferation and metastasis under hypoxic stress (136). Further studies revealed significantly lower expression levels miRNA-569 in PC cells and tissues compared to normal tissues. Experimental evidence has confirmed that miRNA-569 can directly target binding to the 3’-untranslated region (3’-UTR) of *NUSAP1*, thereby inhibiting its expression and reducing the mRNA level of *NUSAP1* by approximately 60%. Moreover, upon overexpression of miRNA-569, the migratory and invasive capabilities of PC cells decreased by approximately 60%, suggesting that miRNA-569 may significantly suppress the metastatic potential of PC cells through the downregulation of *NUSAP1 (* 137). These findings further substantiate the critical role of NUSAP1 overexpression in promoting the migration and invasion of PC cells. It was also reported that *Zinc finger E-box binding homeobox 1 (ZEB1)* participates in cancer progression and metastasis (138). It was observed that miRNA-569 expression exhibits a negative correlation with *ZEB1*. In contrast, *NUSAP1* expression positively correlates with *ZEB1*, suggesting that the miRNA-569/NUSAP1/ZEB1 axis may play an important role in regulating the metastatic and invasive ability of PC cells (137).

An in-depth study demonstrated an interaction between NUSAP1 and lactate dehydrogenase A (LDHA) in pancreatic ductal adenocarcinoma (PDAC). The upregulation of NUSAP1 leads to enhanced LDHA activity, forming a positive feedback loop by promoting glycolysis and lactate production, ultimately inhibiting NUSAP1 degradation. This positive feedback mechanism reinforces the Warburg effect, whereby cancer cells prefer to generate energy through aerobic glycolysis rather than oxidative phosphorylation. The synergistic interaction of NUSAP1 and LDHA significantly enhances the migration and invasion of PDAC cells, which means that the NUSAP1/LDHA axis may be a potential target for therapies against PDAC (139). AMP-activated protein kinase (AMPK) has been proven to suppress tumor growth by inhibiting rapid cell proliferation and arresting the cell cycle (140). Recent studies have discovered that increased NUSAP1 expression promotes the EMT process, a critical step for cancer cells to acquire invasive and migratory capabilities. This enhancement contributes to enhanced proliferation, migration, and invasion of PDAC cells. At the same time, NUSAP1 restricts intracellular energy homeostasis and metabolic regulation by reducing AMPK phosphorylation, thereby promoting cancer cell proliferation and metastasis (141). NUSAP1 plays a central regulatory role in the occurrence, development, and metastasis of PDAC by regulating multiple molecular mechanisms such as cell cycle, metabolism, and EMT process. Its expression level holds potential as a significant biomarker for both the diagnosis and prognosis of PDAC.

### 5.4 Comparison of NUSAP1 with other biomarkers

In recent years, the ongoing discovery and validation of biomarkers have significantly advanced clinical diagnosis, prognosis, and treatment across various diseases. Furthermore, integrating biomarkers with nanotechnology has markedly improved the early detection and diagnosis of diseases, including tumors and neurological disorders (142, 143). Traditional biomarkers for digestive system tumors, such as AFP, CEA, and CA19-9, are widely utilized in clinical settings for diagnosis and prognosis evaluation. Nevertheless, these traditional biomarkers are susceptible to interference from multiple factors, resulting in limited specificity and sensitivity. To align with contemporary trends in clinical diagnostics, novel biomarkers for digestive system tumors continue to be identified (144, 145).

Emerging biomarkers include m5C, long non-coding RNA (lncRNA) NORAD, small nucleolar RNA host gene 16 (SNHG16), and NUSAP1. Among these, the novel biomarker m5C regulates the expression of multiple oncogenes, thereby influencing tumor cell proliferation, migration, invasion, and chemoresistance (146). The lncRNAs NORAD and SNHG16 function as competitive endogenous RNAs (ceRNAs) by sponging microRNAs, thereby constructing lncRNA/miRNA/mRNA regulatory networks that modulate the expression of target genes implicated in tumorigenesis. Additionally, this process is associated with the activation of multiple signaling (147, 148). NUSAP1, as a critical mitotic regulator, exhibits tightly controlled expression levels and subcellular localization during the cell cycle. Therefore, abnormal expression levels of NUSAP1 often lead to abnormal cell proliferation, a characteristic that aligns with the mechanism of cancer occurrence. Moreover, the literature indicates that in the pre-cancerous transformation stage, mitotic dysregulation precedes genomic instability (12–15, 149). Secondly, similar to the aforementioned novel biomarkers, NUSAP1 plays a critical role in regulating multiple signaling pathways and modulating the expression levels of various miRNAs, thereby contributing to the carcinogenic process. This suggests that NUSAP1 can reflect tumor occurrence from multiple perspectives and is highly valuable for early tumor identification. Furthermore, as previously discussed, NUSAP1 promotes chemotherapy resistance by enhancing DNA damage repair and compromises immunotherapy efficacy by facilitating tumor immune escape (104, 105). These findings collectively indicate that NUSAP1 is a promising prognostic biomarker and therapeutic target. Consequently, NUSAP1 is involved in nearly every stage of tumor initiation and progression. Compared with other novel biomarkers, it is associated with a broader spectrum of carcinogenic pathways, making it an excellent candidate for diagnostic and prognostic evaluation, as well as a promising therapeutic target.

## 6 Summary and prospect

NUSAP1, a microtubule-associated protein, plays an essential role in the formation of the mitotic spindle. The precise spatiotemporal regulation of its localization and expression levels throughout the cell cycle is vital to ensure accurate chromosome segregation and proper mitotic progression. Dysregulation of NUSAP1’s ability to maintain stable cell proliferation frequently results in various adverse consequences, including early embryonic lethality and the development and progression of various cancers (12, 14, 15). These findings highlight the potential significance of NUSAP1 in elucidating the pathogenesis of diseases characterized by abnormal cell proliferation.

Research has shown that the expression of NUSAP1 is significantly increased across various types of tumors and is closely associated with tumor invasion, disease advancement, poor prognosis, and treatment outcomes. These findings are consistent with NUSAP1’s inherent pro-proliferative activity. Current research has firmly demonstrated that NUSAP1 exerts regulatory functions in several key signaling pathways, including the Wnt/B-catenin, Hedgehog, PI3K/Akt, and TGF-B pathways (21, 59, 65, 78). Through mechanisms such as DNA repair, cell metabolism modulation, and cell cycle regulation, NUSAP1 facilitates tumor progression. Further experimental and clinical investigations are warranted to elucidate the precise role of NUSAP1 in tumor biology, which may provide novel insights for the treatment of malignant tumors, particularly those of the digestive system.

A substantial body of research has employed diverse bioinformatics approaches to identify oncogenic genes, revealing that *NUSAP1* is a hub gene frequently observed in various digestive system tumors, including hepatocellular carcinoma (85), gastric cancer (122), colorectal cancer (124–127), and pancreatic cancer (131–134). Further analysis using COX risk regression and the construction of prognostic models have demonstrated that the overexpression of *NUSAP1* is associated with poor prognosis in digestive system tumors. These findings suggest potential overlapping pathogenic mechanisms among these malignancies and highlight the critical role of *NUSAP1* within the gene expression network. However, its precise biological functions require comprehensive validation through extensive *in vivo* and *in vitro* experiments for further clarification. Future research should aim to investigate the associations and common mechanisms of NUSAP1 across different digestive system tumors.

The interactions between miRNAs and their regulation of downstream transcription factors are crucial in the development of cancer. Some miRNAs are considered promising immune-related biomarkers with prognostic value and significantly influence the proliferation, migration, invasion, and immune escape mechanisms of certain tumor cells (150). Based on the preceding analysis, it is evident that NUSAP1 serves as an important mediator in the miRNA-regulated progression of digestive system tumors. For instance, in liver cancer patients, decreased expression of miR-193a-5p, miR-122, and miR-18b results in elevated *NUSAP1* levels, which in turn promotes the proliferation of hepatocellular carcinoma cells (97–99). NUSAP1 enhances radioresistance in GC cells by inhibiting ANXA2 ubiquitination. Additionally, miR-129-5p directly targets NUSAP1, and its downregulation significantly increases radiosensitivity in these cells (121). These findings indicate that up-regulating specific miRNAs or inhibiting NUSAP1 expression to target the miRNA/NUSAP1 pathway may represent a promising therapeutic strategy for digestive system tumors (**Table 3**).

**Table 3:** NUSAP1-associated microRNAs and their functions in cancers.

MicroRNAs	Digestive system neoplasms	Mechanism of function	References
miRNA-193a-5p	Liver cancer	Downregulation of miRNA-193a-5p results in increased NUSAP1 expression.	(97, 97)
miRNA-122	Liver cancer	miRNA-122 knockdown leads to increased NUSAP1 expression.	(98, 98)
miRNA-18b	Liver cancer	HBx contributes to higher NUSAP1 expression through the downregulation of miRNA-18b.	(99, 99)
miRNA-129-5p	Gastric cancer	miRNA-129-5p targets NUSAP1 to decrease its expression for enhancing radiotherapy sensitivity.	(121, 121)
miRNA-569	Pancreatic cancer	Promotion of pancreatic cancer cell invasion through the microRNA-569/NUSAP1/ZEB1 axis.	(137, 138)
miRNA-490-3p	Osteosarcoma	miRNA-490-3p mediates the regulation of apoptosis and cell cycle by targeting NUSAP1 and CDCA8/ATF3.	(152)
miRNA-758-3p	Non-small cell lung cancer	miRNA-758-3p inhibits the proliferation, migration, and invasion abilities of NSCLC cells by targeting NUSAP1.	(153)
miRNA-128-3p	Glioblastoma	LINC01393 promotes the occurrence and development of Glioblastoma by up-regulating NUSAP1 as a ceRNA of miRNA-128-3p and activating the NF-κB pathway.	(154)

Moreover, NUSAP1 can modulate downstream transcription factors to activate multiple signaling pathways, thereby inducing cancer cell stemness and contributing to early tumor recurrence. Additionally, NUSAP1 influences the immune microenvironment, facilitating tumor immune escape. Moreover, NUSAP1 is crucial in determining the sensitivity and resistance to chemotherapy drugs; reducing its expression can enhance the sensitivity of cancer cells to chemotherapeutic agents (131). E2F8 transcriptionally activates the target gene *NUSAP1*, thereby alleviating DNA damage and mediating cisplatin resistance in liver cancer cells. Conversely, the knockdown of *NUSAP1* significantly increases DNA damage and restores cisplatin sensitivity in these cells (104). Simultaneously, the high expression of NUSAP1 may suppress immune responses, indicating its potential as a novel immune checkpoint and offering a new avenue for optimizing tumor immunotherapy. NUSAP1 inhibits the differentiation of PBMCs into DCs via the SHCBP1/JAK2/STAT3 pathway, thereby promoting immune escape. Targeted suppression of NUSAP1 not only reinstates the differentiation potential of DCs but also synergistically amplifies the efficacy of immunotherapy (105). Consequently, NUSAP1 holds significant potential for research in specific therapies (such as chemotherapy, radiotherapy, and immunotherapy), efficacy evaluation, postoperative surveillance, and prognosis in patients with digestive system tumors.

Although the structure, function, and role of NUSAP1 in tumors have been extensively investigated, several critical issues warrant further exploration. Post-translational modifications, including phosphorylation, ubiquitination, and SUMOylation, are essential for NUSAP1’s functions in promoting microtubule formation and regulating the cell cycle (31–37). Current studies primarily focus on how NUSAP1 influences tumor progression by modulating the post-translational modifications of other proteins; however, no research has directly established a link between NUSAP1’s own post-translational modifications and tumor progression. This gap represents a promising direction for future research, as elucidating this relationship could facilitate a deeper understanding of NUSAP1’s unique role in tumor development and its potential applications in clinical diagnosis and treatment.

In summary, as research on NUSAP1 and digestive system tumors continues to deepen, the importance of NUSAP1 in various malignant biological behaviors of tumors has become increasingly apparent. NUSAP1 holds promise as a novel tumor biomarker, offering new avenues for clinical diagnosis and prognosis evaluation in digestive system cancers. Additionally, targeted therapies aimed at NUSAP1 may provide more promising treatment options for patients with digestive system tumors.

## Glossary

NUSAP1: Nucleolar spindle-associated protein 1
HCC: Hepatocellular carcinoma
iCCA: Intrahepatic cholangiocarcinoma
TACE: Transcatheter arterial chemoembolization
TKIs: Tyrosine kinase inhibitors
EC: Esophageal cancer
ESCC: Esophageal squamous cell carcinoma
EAC: Esophageal adenocarcinoma
HRQOL: Health-related quality of life
OS: Overall survival
GC: Gastric cancer
CRC: Colorectal cancer
PC: Pancreatic cancer
DFS: Disease-free survival
PDAC: Pancreatic ductal adenocarcinoma
EMT: Epithelial-mesenchymal transition
CSC: Cancer stem cell
TCF/LEF: T-cell factor/lymphoid enhancer factor
GSK-3β: Glycogen synthase kinase-3β
MEF2D: Myocyte enhancer factor 2D
ANKRD22: Ankyrin repeat domain 22
HH: Hedgehog
GLI: Glioma-associated oncogene
PI3K: Phosphoinositide 3-kinase
Akt: Protein kinase B
BTG2: B-cell translocation gene 2
HOXB2: Homeobox protein Hox-B2
mTOR: Mammalian target of rapamycin
TGF-β: Transforming growth factor-beta
TGFBR1: TGF-β receptor type 1
Smad2/3: Mothers against decapentaplegic homolog 2/3
YAP1: Yes-associated protein 1
DNMT1: DNA methyltransferase 1
LDHA: Lactate dehydrogenase A
AMPK: AMP-activated protein kinase ZEB1, Zinc finger E-box binding homeobox 1
SHCBP1: SHC and spindle-associated protein 1
JAK2/STAT3: Janus kinase 2/signal transducer and activator of transcription 3
PBMCs: Peripheral blood mononuclear cells
DCs: Dendritic cells
AFP: Alpha-fetoprotein
CEA: Carcinoembryonic antigen
CA19-9: Carbohydrate antigen 19-9
m5C: 5-Methylcytosine
lncRNA: Long non-coding RNA
NORAD: Non-coding RNA activated by DNA damage
SNHG16: Small nucleolar RNA host gene 16
ceRNA: Competitive endogenous RNA
AI: Artificial intelligence
ML: Machine learning
CDK4/6: Cyclin-dependent kinase 4/6
APC/C: Anaphase-promoting complex/cyclosome
MCAK: Mitotic centromere-associated kinesin
RanGTP: Ras-related nuclear protein GTP
Impα/β/7: Importin alpha/beta/7
SUMO: Small ubiquitin-like modifier
RANBP2: Ran-binding protein 2
KEN box: Lysine-glutamine-asparagine box
ChHD: Conserved high-charge binding domain
SAP: SAF-A/B, Acinus, and PIAS domain
MAPs: Microtubule-associated proteins
MTs: Microtubules
SAC: Spindle assembly checkpoint
PEF: Polar ejection force
SAFs: Spindle assembly factors
LRP5/6: Lipoprotein receptor-related protein 5/6
E2F8: E2F transcription factor 8
RAD51: DNA repair protein RAD51
NF-κB: Nuclear factor kappa-light-chain-enhancer of activated B cells
ATM: Ataxia-telangiectasia mutated
PPARγ: Peroxisome proliferator-activated receptor gamma
CDCA8: Cell division cycle-associated protein 8
ATF3: Activating transcription factor 3
LINC01393: Long intergenic non-protein coding RNA 1393
